# Surviving SARS and living through COVID-19: Healthcare worker mental health outcomes and insights for coping

**DOI:** 10.1371/journal.pone.0258893

**Published:** 2021-11-10

**Authors:** Rima Styra, Laura Hawryluck, Allison Mc Geer, Michelle Dimas, Jack Sheen, Peter Giacobbe, Neil Dattani, Gianni Lorello, Valeria E. Rac, Troy Francis, Peter E. Wu, Wing-Si Luk, Enoch Ng, Jeya Nadarajah, Kaila Wingrove, Wayne L. Gold

**Affiliations:** 1 Centre for Mental Health, University Health Network, Toronto, Ontario, Canada; 2 Intensive Care Medicine, University Health Network, Toronto, Ontario, Canada; 3 Division of Infectious Diseases, Sinai Health System & University Health Network, Toronto, Ontario, Canada; 4 Department of Research, William Osler Health System, Brampton, Ontario, Canada; 5 Institute of Medical Science, University of Toronto, Toronto, Ontario, Canada; 6 Department of Psychiatry, Sunnybrook Health Sciences Centre, Toronto, Ontario, Canada; 7 Department of Emergency Medicine, William Osler Health System, Etobicoke, Ontario, Canada; 8 Department of Anesthesia, University Health Network, Toronto, Ontario, Canada; 9 Peter Munk Cardiac Centre, University Health Network, Toronto, Ontario, Canada; 10 Toronto Health Economics and Technology Assessment (THETA) Collaborative, University of Toronto, Toronto, Ontario, Canada; 11 Division of General Internal Medicine, University Health Network, Toronto, Ontario, Canada; 12 Patient Safety & Quality Improvement, University Health Network, Toronto, Ontario, Canada; 13 Infectious Disease, Markham Stouffville Hospital, Markham, Ontario, Canada; Imam Abdulrahman Bin Faisal University, SAUDI ARABIA

## Abstract

**Objective:**

Explore how previous work during the 2003 Severe Acute Respiratory Syndrome (SARS) outbreak affects the psychological response of clinical and non-clinical healthcare workers (HCWs) to the current COVID-19 pandemic.

**Methods:**

A cross-sectional, multi-centered hospital online survey of HCWs in the Greater Toronto Area, Canada. Mental health outcomes of HCWs who worked during the COVID-19 pandemic and the SARS outbreak were assessed using Impact of Events—Revised scale (IES-R), Generalized Anxiety Disorder scale (GAD-7), and Patient Health Questionnaire (PHQ-9).

**Results:**

Among 3852 participants, moderate/severe scores for symptoms of post- traumatic stress disorder (PTSD) (50.2%), anxiety (24.6%), and depression (31.5%) were observed among HCWs. Work during the 2003 SARS outbreak was reported by 1116 respondents (29.1%), who had lower scores for symptoms of PTSD (P = .002), anxiety (P < .001), and depression (P < .001) compared to those who had not worked during the SARS outbreak. Multivariable logistic regression analysis showed non-clinical HCWs during this pandemic were at higher risk of anxiety (OR, 1.68; 95% CI, 1.19–2.15, P = .01) and depressive symptoms (OR, 2.03; 95% CI, 1.34–3.07, P < .001). HCWs using sedatives (OR, 2.55; 95% CI, 1.61–4.03, P < .001), those who cared for only 2–5 patients with COVID-19 (OR, 1.59; 95% CI, 1.06–2.38, P = .01), and those who had been in isolation for COVID-19 (OR, 1.36; 95% CI, 0.96–1.93, P = .05), were at higher risk of moderate/severe symptoms of PTSD. In addition, deterioration in sleep was associated with symptoms of PTSD (OR, 4.68, 95% CI, 3.74–6.30, P < .001), anxiety (OR, 3.09, 95% CI, 2.11–4.53, P < .001), and depression (OR 5.07, 95% CI, 3.48–7.39, P < .001).

**Conclusion:**

Psychological distress was observed in both clinical and non-clinical HCWs, with no impact from previous SARS work experience. As the pandemic continues, increasing psychological and team support may decrease the mental health impacts.

## Introduction

Fear, anxiety, and distress are natural human reactions to emerging infectious diseases [1–3]. Healthcare workers (HCWs) must deal with potential risks of infection to themselves and their families, possible work interruption or redeployment, and the threat of shortages of personal protective equipment. A survey of physicians and nurses in China and Italy during the current coronavirus-19 disease (COVID-19) pandemic [4, 5] reported significant levels of depression, anxiety, insomnia, and post-traumatic distress similar to the responses seen during the 2003 Severe Acute Respiratory Syndrome (SARS) outbreaks [6–8]. The Greater Toronto Area (GTA) was at the center of the 2003 Canadian SARS outbreak [9, 10]. Healthcare experience during a prior infectious diseases outbreak might heighten or attenuate the emotional response to an emerging infectious disease. For HCWs who worked during the SARS outbreak, a recurrence or intensification of psychological distress may occur in response to exposure to a similar trauma, such as the COVID-19 pandemic.

We conducted this study in the GTA to explore the psychological effects of the COVID-19 pandemic on clinical and non-clinical HCWs, to identify factors that may put HCWs at higher risk of poor mental health outcomes and to assess the impact of work during a previous novel pathogen outbreak, namely the 2003 SARS outbreak in Toronto, on mental health outcomes. As the COVID-19 pandemic continues into its next waves, the results of this study are crucial to assist in the development of strategies to address the mental health needs of HCWs to support their wellbeing, promote their retention and to preserve a high-functioning workforce during this pandemic, and those that will arise in the future.

## Methods

### Study design

This study was a cross-sectional, multi-centered, hospital-based online survey conducted in two tertiary and two community care hospitals. Ethics approval was obtained from the Board of Record assigned from Clinical Trials Ontario for all sites.

Participants were recruited via an internal, non-targeted e-mail or through each hospital’s COVID-19 information updates that contained a link to the online survey. The anonymous survey had a landing page outlining consent for participation. The online survey was available for a 14-day period from 14 May to 28 May 2020 in two centers, from 27 May to 10 June 2020 in the third center, and from 19 June to 3 July 2020 in the fourth center. All centers were treating patients with COVID-19 and all had cared for patients with SARS during the 2003 outbreak.

The survey was adapted from a survey used during the SARS outbreak in 2003 [11] and included demographic information, as well as location, type of work and years of experience as a HCW, care of patients with COVID-19, redeployment status, self-report of sleep disturbance and use of sedatives for sleep, alcohol use, isolation or quarantine status, work during the SARS outbreak, and connection to individuals diagnosed with COVID-19 in the current pandemic or to SARS in the previous outbreak. The following self-report scales were embedded in the survey to evaluate the psychological impact of the COVID pandemic: Impact of Event Scale-Revised (IES-R) [12], Generalized Anxiety Disorder Scale (GAD-7) [13] and Patient Health Questionnaire (PHQ-9) [14].

#### Study population

Eligible participants included all personnel working in the participating hospitals. Personnel were categorized as nurses, physicians, allied health (e.g. pharmacy, physiotherapists, occupational therapists, social work), and non-clinical HCWs (e.g. administrative assistants, researcher staff, environmental services). Units were categorized as high-risk (Emergency Department, Intensive Care Unit, units dedicated to the care of patients with COVID, and units dedicated to the care of patients with SARS in the previous outbreak); low-risk (inpatient units not dedicated to COVID-19 care and ambulatory clinics not directly involved in the care of patients with COVID-19); and indirect risk (administrative, research and educational areas).

The sample size of HCWs was determined using the formula N = Z_α_^2^P(1-P)/d^2^, in which α = 0.05, Zα = 1.96, the estimated acceptable margin of error (d) was 0.1, and population proportion estimate (P) was 0.72 based on a large COVID-19 HCW study [4]. We amplified our sampling size by 50% to gain more completed questionnaires, whereby a sample size of 117 HCW per group was estimated.

#### Outcomes and measures

The primary outcomes were symptoms of post-traumatic stress disorder (PTSD), anxiety and depression as determined by validated instruments. The IES-R is a self-report measure to assess current subjective distress resulting from a traumatic event. It consists of 22 items with a rating scale from 0 to 4. IES-R scores are normal (0–8), mild (9–23), moderate (24–32), and severe (≥33) distress [12, 15, 16]. The 7-item Generalized Anxiety Disorder (GAD-7) scores range from 0–21. Scores are normal (0–4), mild (5–9), moderate (10–14), and severe (15–21) anxiety [13]. The 9-item PHQ-9 is a depression scale, with scores being normal (0–4), mild (5–9), moderate (10–14), and severe (15–21) [14]. We used cut-off scores (IES-R = 24/33, GAD = 10/15, and PHQ = 10/15) for identification of moderate and severe symptoms.

Demographic data were self-reported and included the HCWs’ professional roles, category of institution (tertiary or community care), area of work, age, sex, marital status, education, isolation or quarantine history, deterioration in sleep, and sedative and alcohol use. Staff identified whether they cared for patients with COVID-19 and the number of patients cared for, as well as loss of family, friends, or colleagues to COVID-19. Participants who indicated working during the SARS outbreak were also requested to provide the area of work at that time, the number of patients cared for, isolation or quarantine during that period, and loss of family, friends, or colleagues to SARS.

### Statistical analysis

Analyses were performed using R software v3.6.2 (R Core Team, 2019). The significance level for each analysis was set at α = 0.05, and all tests were 2-tailed. Mental health outcome measures were not normally distributed and are reported as medians with interquartile ranges (IQRs). Comparison of categorical variables across groups was analyzed using Pearson’s Chi square tests. Kruskal-Wallis rank sum tests were used to compare the severity of symptoms between groups. Ranked data of the level of symptoms of PTSD, anxiety, and depression are presented as counts and percentages. Overall domain scores were used for each analysis (IES-R, GAD-7, PHQ-9). Missing data was imputed using a series mean. In cases where >5% of the data were missing, the scale was not included.

Multivariable logistic regression analyses were performed to determine risk factors for symptoms of post-traumatic stress, anxiety, and depression. Sensitivity analyses were carried out to determine that the time frame of survey distribution across the centers did not impact results. Associations were presented as odds ratios (OR) with 95% confidence intervals (CI); medians and interquartile ranges (IQR) were also reported. Models adjusted for age, sex, marital status, professional role, quarantine or isolation status, years of HCW experience, category of institution, deterioration in sleep, use of sedatives and alcohol, and emotional support. Multi-collinearity was assessed using the variance inflation factor and McFadden’s Pseudo-R squared determined model fit.

## Results

### Demographic characteristics

Among the 3852 respondents, there were 1256 nurses (34.1%), 1243 non-clinical staff (28.3%), 1034 allied health staff (28.1%), and 345 physicians (9.4%). Two tertiary care hospitals participated with a total of 1770 participants and two community hospitals participated with a total of 1991 participants.

Overall females comprised 84.2% of participants and 2375 HCWs (64.6%) identified that their work involved contact with patients with COVID-19. Sixteen percent (473 participants) had been required to quarantine and 693 participants (19.3%) had been in isolation for COVID-19. Nearly one-half of participants (1868) had a colleague, family member or friend diagnosed with COVID-19 and of these participants, 344 (18.4%) knew someone who had died of COVID-19. Work during the 2003 SARS outbreak was reported by 1116 respondents (29.1%) (Table 1).

**Table 1:** Demographic and professional characteristics of participants.

Characteristic	Allied Health (N = 1075)	Nurses (N = 1298)	Physicians (N = 357)	Non-Clinical (N = 1122)	Total (N = 3852)
	No. (%)				
**Sex**					
Male	161 (15.7)	111 (9.0)	153 (44.6)	147 (14.4)	572 (15.8)
Female	864 (84.3)	1126 (91.0)	190 (55.4)	875 (85.6)	3055 (84.2)
**Age**					
18–25	47 (4.7)	120 (9.8)	3 (0.9)	53 (5.3)	223 (6.2)
26–35	376 (37.6)	404 (33.0)	81 (23.5)	262 (26.1)	1123 (31.5)
36–45	262 (26.2)	300 (24.5)	118 (34.3)	251 (25.0)	931 (26.1)
46–55	219 (21.9)	229 (18.7)	75 (21.8)	270 (26.9)	793 (22.2)
>55	96 (9.6)	170 (13.9)	67 (19.5)	166 (16.6)	499 (14.0)
**Marital Status**					
Married	563 (54.7)	656 (52.6)	261 (75.7)	552 (53.5)	2032 (55.6)
Unmarried	406 (39.4)	506 (40.6)	77 (22.3)	377 (36.5)	1366 (37.4)
Divorced/Widowed	61 (5.9)	84 (6.7)	7 (2.0)	103 (10.0)	255 (7.0)
**Experience**					
<1 Year	30 (2.9)	49 (3.9)	4 (1.2)	46 (4.4)	129 (3.5)
1–5 Years	270 (26.1)	309 (24.6)	45 (13.1)	218 (21.0)	842 (22.9)
6–10 Years	183 (17.7)	228 (18.2)	87 (25.3)	201 (19.3)	699 (19.0)
11–15 Years	179 (17.3)	177 (14.1)	51 (14.8)	157 (15.1)	564 (15.4)
16–20 Years	134 (13.0)	143 (11.4)	43 (12.5)	125 (12.0)	445 (12.1)
21–25 Years	85 (8.2)	98 (7.8)	41 (11.9)	101 (9.7)	325 (8.9)
>25 Years	152 (14.7)	251 (20.0)	73 (21.2)	192 (18.5)	668 (18.2)
**Education**					
College/University	177 (36.8)	259 (42.6)	13 (9.4)	248 (50.1)	697 (40.5)
Professional/ Graduate	300 (62.4)	341 (56.1)	123 (89.1)	236 (47.7)	1000 (58.1)
**Working During SARS**					
No	781 (73.1)	922 (71.2)	232 (65.2)	786 (70.3)	2721 (70.9)
Yes	287 (26.9)	373 (28.8)	124 (34.8)	332 (29.7)	1116 (29.1)
**Redeployed**					
No	830 (80.7)	959 (76.8)	329 (95.1)	895 (86.4)	3013 (82.3)
Yes	199 (19.3)	290 (23.2)	17 (4.9)	141 (13.6)	647 (17.7)
**Area of Work**					
High Risk^1^	344 (33.0)	512 (40.4)	73 (21.1)	244 (22.9)	1173 (31.6)
Low Risk^2^	258 (24.8)	555 (43.8)	198 (57.2)	203 (19.1)	1214 (32.7)
Indirect Risk^3^	439 (42.2)	199 (15.7)	75 (21.7)	617 (58.0)	1330 (35.8)
**Contact with Patients with COVID-19**					
Contact, but Not Daily	439 (41.0)	341 (26.5)	123 (34.6)	686 (61.4)	1589 (41.5)
Daily Contact	266 (24.9)	437 (33.9)	59 (16.6)	129 (11.5)	891 (23.3)
No Direct Contact	365 (34.1)	510 (39.6)	174 (48.9)	302 (27.0)	1351 (35.3)

^1^High-risk areas include: Emergency Department, Intensive Care Unit, dedicated COVID-19 unit;

^2^Low-risk areas include: inpatient units and ambulatory clinics not dedicated to COVID-19;

^3^Indirect-risk areas include: administrative, research and educational areas.

Sedative use was reported by 490 participants (14.1%) and 898 participants (25.7%) had started or increased their use of alcohol. Of the 2009 HCWs (52.1%) who identified deterioration in sleep, 462 (22.9%) reported using sedatives and 677 (33.6%) reported starting or increasing their use of alcohol. A total of 1200 participants (35%) identified neglecting their own health needs.

### Mental health outcome measures of all respondents

A substantial number of participants experienced moderate or severe symptoms of PTSD (1685 [50.2%]), anxiety (827 [24.6%]), and depression (1059 [31.5%]). Moderate or severe symptoms of PTSD were frequent in all HCW subgroups: nurses (55.9%), non-clinical HCWs (50.6%), allied health (49.1%), and physicians (31.3%) (Table 2).

**Table 2:** Severity categories of anxiety, post-traumatic stress disorder and depression by profession, sex, and hospital type.

Severity of Symptoms	Profession						Sex				Hospitals			
	No. (%)													
	Allied Health (N = 1075)	Nurses (N = 1298)	Physicians (N = 357)	Non-Clinical (N = 1122)	Total (N = 3852)	P Value	Male (N = 572)	Female (N = 3065)	Total (N = 3637)	P Value	Community (N = 1919)	Tertiary (N = 1770)	Total (N = 3689)	P Value
**IES-R, PTSD**						< .001				< .001				< .001
Normal	186 (19.3)	179 (15.5)	108 (33.9)	186 (20.3)	659 (19.6)		150 (28.6)	501 (18.0)	651 (19.6)		387 (22.1)	271 (16.9)	658 (19.6)	
Mild	304 (31.6)	331 (28.6)	111 (34.8)	267 (29.1)	1013 (30.2)		155 (29.6)	845 (30.3)	1000 (30.2)		559 (32.0)	454 (28.3)	1013 (30.2)	
Moderate	163 (16.9)	177 (15.3)	39 (12.2)	151 (16.4)	530 (15.8)		62 (11.8)	463 (16.6)	525 (15.8)		264 (15.1)	264 (16.5)	528 (15.7)	
Severe	310 (32.2)	470 (40.6)	61 (19.1)	314 (34.2)	1155 (34.4)		157 (30.0)	981 (35.2)	1138 (34.3)		539 (30.8)	615 (38.3)	1154 (34.4)	
**GAD-7, Anxiety**						< .001				< .001				< .001
Normal	432 (45.0)	498 (43.4)	200 (61.3)	434 (47.1)	1564 (46.6)		304 (57.3)	1242 (44.6)	1546 (46.7)		916 (51.6)	648 (41.1)	1564 (46.7)	
Mild	295 (30.8)	337 (29.4)	75 (23.0)	257 (27.9)	964 (28.7)		133 (25.0)	826 (29.7)	959 (28.9)		459 (25.9)	503 (31.9)	962 (28.7)	
Moderate	149 (15.5)	195 (17.0)	36 (11.0)	145 (15.7)	525 (15.6)		63 (11.9)	452 (16.2)	515 (15.5)		263 (14.8)	261 (16.6)	524 (15.6)	
Severe	83 (8.7)	118 (10.3)	15 (4.6)	86 (9.3)	302 (9.0)		31 (5.8)	262 (9.4)	293 (8.8)		137 (7.7)	165 (10.5)	302 (9.0)	
**PHQ-9, Depression**						< .001				< .001				< .001
Normal	400 (41.7)	438 (37.9)	194 (59.3)	347 (37.6)	1379 (41.0)		267 (50.4)	1098 (39.3)	1365 (41.1)		810 (45.4)	569 (36.0)	1379 (41.0)	
Mild	268 (27.9)	325 (28.1)	77 (23.5)	258 (28.0)	928 (27.6)		133 (25.1)	787 (28.2)	920 (27.7)		461 (25.9)	464 (29.4)	925 (27.5)	
Moderate	248 (25.8)	306 (26.4)	49 (15.0)	251 (27.2)	854 (25.4)		104 (19.6)	737 (26.4)	841 (25.3)		410 (23.0)	444 (28.1)	854 (25.4)	
Severe	44 (4.6)	88 (7.6)	7 (2.1)	66 (7.2)	205 (6.1)		26 (4.9)	173 (6.2)	199 (6.0)		102 (5.7)	103 (6.5)	205 (6.1)	

IES-R, 22-item Impact of Event Scale-Revised, PTSD: Post-Traumatic Stress Disorder; GAD-7, 7-item Generalized Anxiety Disorder; PHQ-9, 9-item Patient Health Questionnaire.

Staff who were isolated or quarantined during the COVID-19 pandemic scored higher on all 3 metal health outcomes than those who were not required to do so (P < .001) (S1 Table). HCWs reporting use of sedatives experienced proportionally more moderate or severe symptoms of PTSD (81.2%, vs 51.6%, P < .001), anxiety (51.6% vs 19.8%, P < .001), and depression (62.2% vs 25.9%, P < .001) compared to HCWs who did not use sleep medications, which was similar for those who started or increased their use of alcohol during the pandemic (S2 and S3 Tables). More than one-third of HCW’s reported neglecting their own health and these HCWs were more likely to report greater symptoms of PTSD, anxiety, and depression compared to those who did not report neglecting their own health (P < .001) (S4 Table).

### The impact of previous work during SARS

Those who worked during the SARS outbreak experienced lower scores of post-traumatic stress symptoms (median, 21.00 vs. 24.00, P = .002), anxiety (median, 4.00 vs 5.00, P < .001); and depression (median, 5.00 vs 6.00, P < .001) compared to those who had not worked during SARS (Table 3). Regarding the 2003 SARS outbreak, participants who had worked in high-risk areas, cared for patients with SARS, or knew someone who was infected or had died of SARS did not experience significant differences in mental health outcomes during the present COVID-19 pandemic compared to those who had not (Table 4). HCWs who were isolated or quarantined during the SARS outbreak (31/1082 [2.8%]), compared to those who were not, reported higher overall levels of post-traumatic stress symptoms (median, 36.00 [IQR, 18.75–48.25]) vs. median, 20.50 [IQR, 10.0–37.0] P = .002): avoidance (P = .04), intrusive symptoms (P < .001) and hyperarousal symptoms (P < .001), as well as symptoms of anxiety (P = .013). We proceeded to a multivariable analysis which adjusted for such variables as age, gender, marital status, profession, quarantine/isolation, years of experience, use of sedatives, started/increased alcohol use, hospital type, as well as SARS experience. In this analysis, no significant odds ratios were found for those who had worked during SARS and those who had not worked during that time with regards to mental health outcomes.

**Table 3:** Mental health outcomes of healthcare workers who had and had not worked during the 2003 Severe Acute Respiratory Syndrome outbreak.

Outcomes	Worked During SARS		
	No (N = 2726)	Yes (N = 1116)	P Value
**IES-R, Avoidance**			< .001
Median	9.00	8.00	
Q1, Q3	4.00, 15.00	3.00, 12.00	
Range	0.00–32.00	0.00–32.00	
**IES-R, Intrusive**			.03
Median	9.00	8.00	
Q1, Q3	4.00, 16.00	4.00, 14.00	
Range	0.00–32.00	0.00–32.00	
**IES-R, Hyperarousal**			.04
Median	6.00	5.00	
Q1, Q3	2.00, 11.00	2.00, 10.00	
Range	0.00–24.00	0.00–24.00	
**IES-R Total**			.002
Median	24.00	21.00	
Q1, Q3	11.00, 40.00	11.00, 37.00	
Range	0.00–88.00	0.00–88.00	
**GAD-7 Total**			< .001
Median	5.00	4.00	
Q1, Q3	2.00, 10.00	1.00, 8.00	
Range	0.00–21.00	0.00–21.00	
**PHQ-9 Total**			< .001
Median	6.00	5.00	
Q1, Q3	2.00, 12.00	2.00, 11.00	
Range	0.00–27.00	0.00–27.00	

SARS: Severe Acute Respiratory Syndrome; IES-R: 22-item Impact of Event Scale-Revised; GAD-7: 7-item Generalized Anxiety Disorder; PHQ-9: 9-item Patient Health Questionnaire; Q1: 1^st^ Quartile median score; Q3: 3^rd^ Quartile median score.

**Table 4:** Work experience during SARS and mental health outcomes in healthcare workers.

Outcomes	Isolated/Quarantined			Risk Area of Work				Care of Patients with SARS			Knew Someone Infected or Who Died of SARS		
	No (N = 1065)	Yes (N = 31)	P Value	High Risk^1^ (N = 402)	Low Risk^2^ (N = 436)	Indirect Risk^3^ (N = 270)	P Value	No (N = 794)	Yes (N = 300)	P Value	No (N = 630)	Yes (N = 463)	P Value
**IES-R Avoidance**			.04				.62			.21			.49
Median	8.00	10.00		8.00	8.00	7.00		7.00	8.00		8.00	8.00	
Q1, Q3	3.00, 12.00	6.00, 13.25		3.00, 13.00	3.00, 12.25	3.00, 11.00		3.00, 12.00	3.00, 13.25		3.00, 12.00	4.00, 13.00	
Range	0.00–32.00	3.00–21.00		0.00–31.00	0.00–32.00	0.00–32.00		0.00–32.00	0.00–32.00		0.00–31.00	0.00–32.00	
**IES-R Intrusive**			< .001				.08			.10			.02
Median	8.00	13.50		9.00	8.00	7.00		8.00	9.00		7.00	9.00	
Q1, Q3	3.00, 14.00	9.25, 20.50		4.00, 16.00	4.00, 14.00	3.00, 12.75		3.00, 14.00	4.00, 16.00		3.00, 14.00	4.00, 15.00	
Range	0.00–32.00	1.00–30.00		0.00–30.00	0.00–32.00	0.00–32.00		0.00–32.00	0.00–32.00		0.00–32.00	0.00–32.00	
**IES-R Hyper**			.001				.31			.13			.13
Median	5.00	10.50		6.00	5.00	5.00		5.00	6.00		5.00	5.50	
Q1, Q3	2.00, 10.00	4.75, 15.25		2.00, 11.00	2.00, 9.00	2.00, 10.00		2.00, 10.00	2.00, 12.00		2.00, 10.00	2.00, 11.00	
Range	0.00–24.00	1.00–24.00		0.00–24.00	0.00–24.00	0.00–24.00		0.00–24.00	0.00–24.00		0.00–24.00	0.00–24.00	
**IES-R Total**			.002				.24			.12			.12
Median	20.50	36.00		22.00	20.00	20.00		20.00	24.00		20.00	22.00	
Q1, Q3	10.00, 37.00	18.75, 48.25		11.00, 39.00	11.00, 35.00	9.00, 34.00		11.00, 36.00	11.00, 39.00		10.00, 35.00	11.00, 38.00	
Range	0.00–88.00	7.00–73.00		0.00–82.00	0.00–82.00	0.00–88.00		0.00–88.00	0.00–82.00		0.00–82.00	0.00–88.00	
**GAD-7 Total**			.013				.70			.12			.95
Median	4.00	6.50		4.00	4.00	4.00		4.00	5.00		4.00	4.00	
Q1, Q3	1.00, 8.00	4.75, 10.25		1.00, 8.00	1.00, 9.00	1.00, 7.00		1.00, 8.00	1.00, 9.25		1.00, 8.00	1.00, 8.50	
Range	0.00–21.00	0.00–21.00		0.00–21.00	0.00–21.00	0.00–21.00		0.00–21.00	0.00–21.00		0.00–21.00	0.00–21.00	
**PHQ-9 Total**			.09				.42			.14			.85
Median	5.00	6.00		6.00	6.00	4.00		5.00	6.00		5.00	5.50	
Q1, Q3	2.00, 10.00	3.75, 12.25		1.00, 11.00	2.00, 10.00	2.00, 10.00		2.00, 10.00	1.00, 12.00		2.00, 10.00	2.00, 11.00	
Range	0.00–27.00	0.00–26.00		0.00–26.00	0.00–27.00	0.00–26.00		0.00–27.00	0.00–26.00		0.00–27.00	0.00–26.00	

GAD-7: 7-item Generalized Anxiety Disorder; IES-R, 22-item Impact of Event Scale-Revised; PHQ-9: 9-item Patient Health Questionnaire; SARS: Severe Acute Respiratory Syndrome; HCWs: healthcare workers; Q1: 1^st^ Quartile median score; Q3: 3^rd^ Quartile median score.

^1^High-risk areas include: Emergency Department, Intensive Care Unit, COVID-19 unit;

^2^Low-risk areas include: inpatient units and ambulatory clinics not dedicated to COVID-19;

^3^Indirect-risk areas include: administrative, research and educational areas.

### Risk factors and odds ratios for mental health outcomes

Multivariable logistic regression analysis showed that after controlling for confounders, particular risk factors were identified for symptoms of post-traumatic stress, anxiety, and depression, as assessed using self-report questionnaires of IES-R, GAD-7, and PHQ-9, respectively. Those using sedatives (OR, 2.55; 95% CI, 1.61–4.03, P < .001), those who cared for only 2–5 patients with COVID-19 (OR, 1.59; 95% CI, 1.06–2.38, P = .01), and those who had been in isolation for COVID-19 (OR, 1.36; 95% CI, 0.96–1.93, P = .05), were at higher risk of post-traumatic stress symptoms (Table 5). Non-clinical HCWs were at higher risk of symptoms of anxiety (OR, 1.96; 95% CI, 1.39–2.76, P < .001) and depression (OR, 2.03; 95% CI, 1.34–3.07, P < .001) than other categories of HCWs; whereas physicians were at lower risk of symptoms of PTSD (OR, 0.46; 95% CI, 0.25–0.83, P = .01) and depression (OR, 0.46; 95% CI, 0.22–0.99, P = .01). Unmarried individuals (OR, 1.55; 95% CI, 1.12–2.35, P = .001) had a higher risk of anxiety and depression. New or increased alcohol use was associated with elevated risk on measures of post-traumatic stress symptoms (OR, 1.96; 95% CI, 1.39–2.76, P < .001), anxiety (1.68; 95% CI, 1.19–2.35, P = 0.001), and depression (OR, 2.02; 95% CI, 1.37–2.97, P<0.001). In addition, deterioration in sleep was significantly associated with symptoms of PTSD (OR, 4.68, 95% CI, 3.74–6.30, P <0.001), anxiety (OR, 3.09; 95% CI, 2.11–4.53, P <0.001), and depression (OR 5.07; 95% CI, 3.48–7.39, P <0.001). Working in tertiary care centers was associated with an elevated risk of post-traumatic stress symptoms (OR, 1.88; 95% CI, 1.39–2.54, P<0.001)) and depression (OR, 1.32; 95% CI, 0.96–1.81, P<0.001). Greater years of experience was associated with lower post-traumatic stress symptoms (OR, 1.59; 95% CI, 1.06–2.38, P<0.01), and HCW who were over 45 years of age (OR, 0.54; 95% CI, 0.35–0.81, P<0.001) and ages 60 and above (OR, 0.40; 95% CI, 0.22–0.71, P<0.001) were at lower risk for anxiety. HCWs who reported that they were managing without professional mental health were less likely to experience post-traumatic stress symptoms (OR, 0.31; 95% CI, 0.21–0.44, P<0.001), anxiety (OR, 0.24; 95% CI, 0.17–0.34, P<0.001), and depression (OR, 0.31; 95% CI, 0.22–0.43, P<0.001).

**Table 5:** Multivariable logistic regression analysis of identified risk factors for mental health outcomes.

Outcomes	Moderate & Severe Symptom (N)/Total Number	Adjusted OR (95% CI)	P Value
**IES-R, PTSD**			
**COVID Patients Cared For**			
None	709 / 1584	1	
1	116 / 227	0.98 (0.54–1.77)	.95
2–5	323 / 602	1.59 (1.06–2.38)	.01
6+	528 / 931	1.30 (0.90–1.88)	.16
**Profession**			
Allied Health	473 / 963	1	
Nurses	647 / 1157	1.11 (0.78–1.58)	.56
Physicians	100 / 319	0.46 (0.25–0.83)	.01
Non-Clinical	465 / 918	1.26 (0.86–1.85)	.67
**Placed in Isolation**			
No	1289 / 2733	1	
Yes	227 / 623	1.36 (0.96–1.93)	.05
**Sedative Use**			
No	1217 / 2755	1	
Yes	384 / 473	2.55 (1.61–4.03)	< .001
**Started/Increased Alcohol Use**			
No	1034 / 2384	1	
Yes	584 / 872	1.96 (1.39–2.76)	< .001
**Institution Type**			
Community	803 / 1749	1	
Tertiary Care	879 / 1604	1.88 (1.39–2.54)	< .001
**Experience**			
<1 Year	58 / 112	1	
1–5 Years	421 / 764	0.68 (0.32–1.47)	.33
6–16 Years	589 / 1153	0.55 (0.26–1.17)	.12
17+ Years	613 / 1321	0.37 (0.17–0.84)	.01
**Sleep Quality Deteriorated**			
No	323 / 1411	1	
Yes	1359 / 1940	4.68 (3.74–6.30)	< .001
**Managing Without Professional Mental Health Support**			
No	596 / 761	1	
Yes	1425 / 2414	0.31 (0.21–0.44)	< .001
**GAD-7, Anxiety**			
**Sex**			
Male	94 / 531	1	
Female	714 / 2782	1.59 (0.97–2.62)	.05
**Age**			
18–45	575 / 2066	1	
46–59	149 / 730	0.54 (0.35–0.81)	.001
60+	81 / 461	0.40 (0.22–0.71)	.001
**Marital Status**			
Married	461 / 2101	1	
Unmarried	357 / 1230	1.55 (1.12–2.15)	.001
**Profession**			
Allied Health	232 / 959	1	
Nurses	313 / 1148	1.11 (0.75–1.64)	.60
Physicians	51 / 326	0.96 (0.47–1.96)	.92
Non-clinical	231 / 922	1.68 (1.09–2.59)	.01
**Quarantined**			
No	653 / 2752	1	
Yes	133 / 440	1.48 (0.98–2.24)	.05
**Started/Increased Alcohol**			
No	475 / 2376	1	
Yes	319 / 868	1.68 (1.19–2.35)	.001
**Sleep Quality Deteriorated**			
No	122 / 1406	1	
Yes	704 / 1916	3.09 (2.11–4.53)	< .001
**Managing Without Professional Mental Health Support**			
No	395 / 757	1	
Yes	397 / 2389	0.24 (0.17–0.34)	< .001
**PHQ-9, Depression**			
**Marital Status**			
Married	585 / 2112	1	
Unmarried	464 / 1231	1.65 (1.19–2.27)	.001
**Profession**			
Allied Health	292 / 960	1	
Nurses	394 / 1157	1.07 (0.74–1.56)	.71
Physicians	56 / 327	0.46 (0.22–0.99)	.01
Non-clinical	317 / 922	2.03 (1.34–3.07)	< .001
**Sedative Use**			
No	715 / 2761	1	
Yes	290 / 466	2.02 (1.37–2.97)	< .001
**Started/Increased Alcohol**			
No	617 / 2386	1	
Yes	404 / 869	1.72 (1.23–2.41)	.001
**Institution**			
Community	512 / 1783	1	
Tertiary	547 / 1580	1.32 (0.96–1.81)	.05
**Sleep Quality Deteriorated**			
No	134 /1409	1	
Yes	923 / 1923	5.07 (3.48–7.39)	< .001
**Managing Without Professional Mental Health Support**			
No	464 / 760	1	
Yes	540 / 2397	0.31 (0.22–0.43)	< .001

GAD-7, 7-item Generalized Anxiety Disorder; IES-R, 22-item Impact of Event Scale-Revised; PHQ-9, 9-item Patient Health Questionnaire; PTSD: Post-Traumatic Stress Disorder.

## Discussion

Participants in our survey reported a high prevalence of moderate or severe symptoms of post-traumatic stress, anxiety, and depression in all HCWs in the current pandemic similar to other studies examining psychological distress [4, 5, 17]. To our knowledge our study is the first to examine the effects of previous work experience during an outbreak of a novel pathogen. The results are both surprising and encouraging: previous work experience during the SARS outbreak did not result in increased psychological distress in HCWs during the COVID-19 pandemic compared to those without prior outbreak experience. These results are important as they can provide insights into how familiarity with outbreak management may temper negative mental health effects and potentially promote staff retention.

Similar to our previous research in SARS [11], caring for fewer patients resulted in greater symptoms of post-traumatic stress. We hypothesize that HCWs caring for a greater number of patients, especially in dedicated COVID-19 or ICU units, may gain a sense of normalization in their work environments. They may receive more extensive education and training, develop standard operating procedures, and build team resilience related to the bonds formed between team members who are jointly working during the pandemic [18]. Our results suggest a greater burden of symptoms of PTSD are experienced by HCWs who cared for only a few patients which may relate to having less confidence and experience with the pandemic and its control measures, and seems to suggest that increased experience may result in a sense of self-efficacy that comes with having experienced a previous outbreak, adding to the resilience of the individual [19, 20]. The finding that those who have experienced a previous outbreak or pandemic had lower levels of distress suggests that the experience of surviving an outbreak physically unscathed may add to the HCWs confidence in precautionary measures and their training. As the COVID-19 pandemic continues, these results may indicate that HCWs growing experience in caring for patients will provide some protection against adverse mental health outcomes. Yet as the pandemic continues, or with future pandemics of emerging infectious diseases, ways to mitigate distress could be accomplished by creating a buddy system with a more experienced or resilient colleague, using simulation-based training prior to clinical duties, and the creation of a team of experienced HCWs to onboard redeployed staff to bolster their internal locus of control.

HCWs who during the COVID-19 pandemic were in isolation were at increased risk of PTSD symptoms, whereas those who were quarantined were at increased risk of symptoms of anxiety. Both of these findings suggest that a personal health experience with COVID-19 is a psychologically traumatic event. In our study, there was a small number of participants who were either isolated or quarantined during SARS, and this group endorsed more symptoms of PTSD, anxiety, and depression than others who had also worked during the SARS outbreak. This is consistent with the significant impact of isolation and quarantine on mental health seen in previous research [1, 21, 22]. Individuals experiencing isolation or quarantine are at increased risk of poorer mental health outcomes, and these effects may be longer lasting or may place the individual at risk of exacerbation when faced with the potential of repeated quarantine or isolation during a future outbreak or pandemic [23]. Since resources for support are often limited, priority for HCWs who were isolated or quarantined should be considered. These individuals may be more vulnerable to distress or feel a greater lack of control and should be flagged for “check-ins” with occupational health or be offered psychological support resources through individual telehealth or online groups both during and after isolation and quarantine.

Research during the COVID-19 pandemic has focused on the experience of nurses and physicians. Allied HCWs and non-clinical staff who form an integral and important part of the healthcare system have not consistently been included. Our study identified that non-clinical HCWs were found to have the greatest burden of symptoms of anxiety and depression in comparison to other HCWs. Reports during the COVID-19 pandemic from Singapore have also demonstrated a higher prevalence of anxiety amongst non-clinical HCWs in comparison to clinical HCWs [24]. Studies of HCWs in the United States found that non-clinical HCWs had a higher risk of depression, anxiety, PTSD, and alcohol use [25], and higher levels of stress [26]. Non-clinical staff may experience less control over their work situations and lower self-efficacy as it relates to core medical knowledge and receive less dedicated education relating to the pandemic compared to clinical HCWs which may account for this observed difference. It is imperative for healthcare institutions to not overlook the need for strong communication, educational interventions, and psychological support for all categories of HCWs, both clinical and non-clinical [27, 28].

A proportion of HCWs endorsed a deterioration in their sleep and reported new or increased use of alcohol and sedatives. Sleep disturbances are common diagnostic symptoms of PTSD as well as depression and may signal that emotional functioning and wellbeing are impacted [29]. An additional concern is the substantial proportion of participants who identified neglecting their own physical health. Sleep hygiene, optimizing sleep habits, relaxation techniques and respect for off-hours time should be promoted by healthcare organizations [30]. Initiating or increasing alcohol consumption has long been recognized as a sign of distress. Educational programs promoting self-awareness and a focus on self-care are essential. Neglect of one’s physical health may result in both poor mental and physical health outcomes that can result in absenteeism and retention issues in the workforce. Institutions may look to address these concerns with more easily accessible on-site healthcare, resources to encourage healthy eating and activity, and dedicated respite centers for HCWs.

This study has several limitations. First, in an effort to broaden the scope of participants, our non-targeted email link did not permit us to estimate the response rate since we are unaware of the number of HCWs who saw the notice and then opted not to participate. Second, due to logistical issues, participation in one center was delayed, which may have resulted in a differential exposure to the pandemic by HCWs. Sensitivity analyses did not suggest this to be the case. The sample selection and size of this cohort may have been affected by the possibility that some of the staff working during SARS have since left the workforce over the past 17 years. This may have been due to retirement or possibly worse mental or physical health outcomes as a result of their experience during SARS. Finally, while community and tertiary care hospitals were included, the survey did not include an environmental scan of mitigating or exacerbating factors at individual organizations, including dedicated resources and leadership interventions, to determine possible differences between the two settings.

## Conclusion

The results of this study demonstrate that HCWs with previous work experience during the SARS outbreak did not have worse mental health outcomes compared to those without any previous experience. These findings have significant implications for staff wellness, the prevention of burnout and promotion and maintenance of staff retention—all of which are ongoing challenges in this current and in future pandemics. Our findings provide guidance for healthcare systems seeking to provide appropriate, targeted, and timely support to HCWs especially those at greater risk, in order to promote individual wellness and a healthy workforce.

## Supporting information

S1 Table Mental health outcomes and required isolation/quarantine during COVID-19. GAD-7: 7-item Generalized Anxiety Disorder; IES-R, 22-item Impact of Event Scale-Revised; PHQ-9: 9-item Patient Health Questionnaire.
(DOCX)

S2 Table Severity symptom categories and use of sedatives. GAD-7: 7-item Generalized Anxiety Disorder; IES-R, 22-item Impact of Event Scale-Revised; PHQ-9: 9-item Patient Health Questionnaire.
(DOCX)

S3 Table Severity symptom categories and alcohol use. GAD-7: 7-item Generalized Anxiety Disorder; IES-R, 22-item Impact of Event Scale-Revised; PHQ-9: 9-item Patient Health Questionnaire.
(DOCX)

S4 Table Mental health outcomes and neglecting their own health. GAD-7: 7-item Generalized Anxiety Disorder; IES-R, 22-item Impact of Event Scale-Revised; PHQ-9: 9-item Patient Health Questionnaire.
(DOCX)
