# Supplementary material for: Surviving SARS and living through COVID-19: Healthcare worker mental health outcomes and insights for coping
Source: PLoS One. 2021 Nov 10;16(11):e0258893. doi: 10.1371/journal.pone.0258893 (PMC8580217; doi:10.1371/journal.pone.0258893)
Supplement: S1 Table — GAD-7: 7-item Generalized Anxiety Disorder; IES-R, 22-item Impact of Event Scale-Revised; PHQ-9: 9-item Patient Health Questionnaire. (DOCX) [file pone.0258893.s001.docx]

**S1 Table. Mental Health Outcomes and Required Isolation/Quarantine during COVID-19**

| **Outcomes** | **Isolation** | | | **Quarantine** | | |
| --- | --- | --- | --- | --- | --- | --- |
|  | **No (N=2933)** | **Yes (N=653)** | **P Value** | **No (N=2944)** | **Yes (N=473)** | **P Value** |
| **IES-R Avoidance** |  |  | < .001 |  |  | < .001 |
| Median | 8.00 | 10.00 |  | 8.00 | 10.00 |  |
| Q1, Q3 | 3.00, 14.00 | 6.00, 15.25 |  | 4.00, 14.00 | 5.00, 15.00 |  |
| Range | 0.00 - 32.00 | 0.00 - 31.00 |  | 0.00 - 32.00 | 0.00 - 31.00 |  |
| **IES-R Intrusive** |  |  | < .001 |  |  | < .001 |
| Median | 8.00 | 11.00 |  | 8.00 | 11.00 |  |
| Q1, Q3 | 3.00, 14.50 | 6.00, 18.00 |  | 4.00, 15.00 | 6.00, 17.00 |  |
| Range | 0.00 - 32.00 | 0.00 - 32.00 |  | 0.00 - 32.00 | 0.00 - 32.00 |  |
| **IES-R Hyper** |  |  | < .001 |  |  | < .001 |
| Median | 5.00 | 8.00 |  | 5.00 | 7.00 |  |
| Q1, Q3 | 2.00, 10.00 | 4.00, 12.00 |  | 2.00, 10.00 | 3.00, 12.00 |  |
| Range | 0.00 - 24.00 | 0.00 - 24.00 |  | 0.00 - 24.00 | 0.00 - 24.00 |  |
| **IES-R Total** |  |  | < .001 |  |  | < .001 |
| Median | 22.00 | 31.00 |  | 23.00 | 29.00 |  |
| Q1, Q3 | 10.00, 38.00 | 16.00, 44.00 |  | 11.00, 38.00 | 15.50, 43.00 |  |
| Range | 0.00 - 88.00 | 0.00 - 85.00 |  | 0.00 - 88.00 | 0.00 - 86.00 |  |
| **GAD-7 Total** |  |  | < .001 |  |  | .004 |
| Median | 5.00 | 6.00 |  | 5.00 | 6.00 |  |
| Q1, Q3 | 1.00, 9.00 | 3.00, 12.00 |  | 1.00, 9.00 | 2.00, 11.00 |  |
| Range | 0.00 - 21.00 | 0.00 - 21.00 |  | 0.00 - 21.00 | 0.00 - 21.00 |  |
| **PHQ-9 Total** |  |  | < .001 |  |  | < .001 |
| Median | 6.00 | 8.00 |  | 6.00 | 7.00 |  |
| Q1, Q3 | 2.00, 11.00 | 4.00, 13.00 |  | 2.00, 11.00 | 3.00, 13.00 |  |
| Range | 0.00 - 27.00 | 0.00 - 27.00 |  | 0.00 - 27.00 | 0.00 - 27.00 |  |

GAD-7: 7-item Generalized Anxiety Disorder; IES-R, 22-item Impact of Event Scale-Revised; PHQ-9: 9-item Patient Health Questionnaire
