# Supplementary material for: Surviving SARS and living through COVID-19: Healthcare worker mental health outcomes and insights for coping
Source: PLoS One. 2021 Nov 10;16(11):e0258893. doi: 10.1371/journal.pone.0258893 (PMC8580217; doi:10.1371/journal.pone.0258893)
Supplement: S2 Table — GAD-7: 7-item Generalized Anxiety Disorder; IES-R, 22-item Impact of Event Scale-Revised; PHQ-9: 9-item Patient Health Questionnaire. (DOCX) [file pone.0258893.s002.docx]

**S2 Table. Severity Symptom Categories and Use of Sedatives**

| **Outcomes** | **Sedative Use** | | | |
| --- | --- | --- | --- | --- |
|  | **No (N=2943)** | **Yes (N=507)** | **Total (N=3450)** | **P Value** |
| **IES-R** |  |  |  | < .001 |
| Normal | 629 (22.8) | 10 (2.1) | 639 (19.8) |  |
| Mild | 909 (33.0) | 79 (16.7) | 988 (30.6) |  |
| Moderate | 430 (15.6) | 70 (14.8) | 500 (15.5) |  |
| Severe | 787 (28.6) | 314 (66.4) | 1101 (34.1) |  |
| **GAD-7** |  |  |  | < .001 |
| Normal | 1422 (51.7) | 90 (19.4) | 1512 (47.0) |  |
| Mild | 785 (28.5) | 135 (29.0) | 920 (28.6) |  |
| Moderate | 364 (13.2) | 134 (28.8) | 498 (15.5) |  |
| Severe | 181 (6.6) | 106 (22.8) | 287 (8.9) |  |
| **PHQ-9** |  |  |  | < .001 |
| Normal | 1279 (46.3) | 59 (12.7) | 1338 (41.5) |  |
| Mild | 767 (27.8) | 117 (25.1) | 884 (27.4) |  |
| Moderate | 603 (21.8) | 208 (44.6) | 811 (25.1) |  |
| Severe | 112 (4.1) | 82 (17.6) | 194 (6.0) |  |

GAD-7: 7-item Generalized Anxiety Disorder; IES-R, 22-item Impact of Event Scale-Revised; PHQ-9: 9-item Patient Health Questionnaire
