# Supplementary material for: Surviving SARS and living through COVID-19: Healthcare worker mental health outcomes and insights for coping
Source: PLoS One. 2021 Nov 10;16(11):e0258893. doi: 10.1371/journal.pone.0258893 (PMC8580217; doi:10.1371/journal.pone.0258893)
Supplement: S3 Table — GAD-7: 7-item Generalized Anxiety Disorder; IES-R, 22-item Impact of Event Scale-Revised; PHQ-9: 9-item Patient Health Questionnaire. (DOCX) [file pone.0258893.s003.docx]

**S3 Table. Severity Symptom Categories and Alcohol Use**

| **Outcomes** | **Alcohol use** | | | |
| --- | --- | --- | --- | --- |
|  | **No (N=2556)** | **Yes (N=925)** | **Total (N=3481)** | **P Value** |
| **IES-R** |  |  |  | < .001 |
| Normal | 570 (23.9) | 74 (8.5) | 644 (19.8) |  |
| Mild | 780 (32.7) | 214 (24.5) | 994 (30.5) |  |
| Moderate | 352 (14.8) | 165 (18.9) | 517 (15.9) |  |
| Severe | 682 (28.6) | 419 (48.1) | 1101 (33.8) |  |
| **GAD-7** |  |  |  | < .001 |
| Normal | 1257 (52.9) | 259 (29.8) | 1516 (46.7) |  |
| Mild | 644 (27.1) | 290 (33.4) | 934 (28.8) |  |
| Moderate | 318 (13.4) | 189 (21.8) | 507 (15.6) |  |
| Severe | 157 (6.6) | 130 (15.0) | 287 (8.8) |  |
| **PHQ-9** |  |  |  | < .001 |
| Normal | 1139 (47.7) | 202 (23.2) | 1341 (41.2) |  |
| Mild | 630 (26.4) | 263 (30.3) | 893 (27.4) |  |
| Moderate | 510 (21.4) | 320 (36.8) | 830 (25.5) |  |
| Severe | 107 (4.5) | 84 (9.7) | 191 (5.9) |  |

GAD-7: 7-item Generalized Anxiety Disorder; IES-R, 22-item Impact of Event Scale-Revised; PHQ-9: 9-item Patient Health Questionnaire
