# Supplementary material for: Surviving SARS and living through COVID-19: Healthcare worker mental health outcomes and insights for coping
Source: PLoS One. 2021 Nov 10;16(11):e0258893. doi: 10.1371/journal.pone.0258893 (PMC8580217; doi:10.1371/journal.pone.0258893)
Supplement: S4 Table — GAD-7: 7-item Generalized Anxiety Disorder; IES-R, 22-item Impact of Event Scale-Revised; PHQ-9: 9-item Patient Health Questionnaire. (DOCX) [file pone.0258893.s004.docx]

**S4 Table. Mental Health Outcomes and Neglecting Their Own Health**

| **Outcomes** | **Neglecting Own Health** | | |
| --- | --- | --- | --- |
|  | **No (N=2254)** | **Yes (N=1200)** | **P Value** |
| **IES-R Avoidance** |  |  | < .001 |
| Median | 7.00 | 11.00 |  |
| Q1, Q3 | 3.00, 12.00 | 7.00, 16.00 |  |
| Range | 0.00 - 32.00 | 0.00 - 32.00 |  |
| **IES-R Intrusive** |  |  | < .001 |
| Median | 7.00 | 13.00 |  |
| Q1, Q3 | 3.00, 12.00 | 7.00, 19.00 |  |
| Range | 0.00 - 32.00 | 0.00 - 32.00 |  |
| **IES-R Hyper** |  |  | < .001 |
| Median | 4.00 | 9.00 |  |
| Q1, Q3 | 1.00, 8.00 | 5.00, 14.00 |  |
| Range | 0.00 - 24.00 | 0.00 - 24.00 |  |
| **IES-R Total** |  |  | < .001 |
| Median | 18.00 | 34.00 |  |
| Q1, Q3 | 8.00, 32.00 | 21.00, 48.00 |  |
| Range | 0.00 - 88.00 | 0.00 - 88.00 |  |
| **GAD-7 Total** |  |  | < .001 |
| Median | 4.00 | 8.00 |  |
| Q1, Q3 | 1.00, 7.00 | 4.00, 14.00 |  |
| Range | 0.00 - 21.00 | 0.00 - 21.00 |  |
| **PHQ-9 Total** |  |  | < .001 |
| Median | 4.00 | 10.00 |  |
| Q1, Q3 | 1.00, 8.00 | 6.00, 16.00 |  |
| Range | 0.00 - 27.00 | 0.00 - 27.00 |  |

GAD-7: 7-item Generalized Anxiety Disorder; IES-R, 22-item Impact of Event Scale-Revised; PHQ-9: 9-item Patient Health Questionnaire
